# A comprehensive investigation into EEG spectral analysis for ADHD brain dominance

**DOI:** 10.3934/Neuroscience.2026017

**Published:** 2026-07-08

**Authors:** Vandana Joshi, Nirali Nanavati

**Affiliations:** Computer Engineering Department, Sarvajanik College of Engineering & Technology, Surat, Gujarat, India

**Keywords:** attention deficit hyperactivity disorder (ADHD), electroencephalography (EEG), spectral analysis, brain dominance

## Abstract

Attention Deficit Hyperactivity Disorder (ADHD) is a neurodevelopmental disorder that significantly impacts the cognitive functions and behaviour of individuals. It is characterized by abnormal brain lateralization in key brain regions linked to attention and cognitive control. This study aimed to explore and analyze the brainwave patterns linked to left and right brain dominance in ADHD, ADHD subtype, and control group using electroencephalography (EEG) spectral features, which have not been investigated in prior studies. Our study examined variations in alpha, beta, theta, and other frequency bands between the brain's left and right hemispheres, employing methodologies like EEG recordings with topographical views and statistical tests. The Asymmetric Index findings suggest a differential power spectrum density (PSD) pattern in ADHD subjects, with a higher average PSD in the right brain (RB) during working states and in the left brain (LB) during resting states. In addition, our corroborative study also uncovered that in ADHD subjects, theta and alpha waves are more dominant in the left hemispheric region, irrespective of the task state, and theta waves are notably prominent in the frontal region. The results of our novel study, supported by statistical analyses and topographical views, not only enhance our understanding of ADHD but also open avenues for more targeted and effective approaches in diagnosing and understanding the neurobiological underpinnings of ADHD and its subtypes.

## 1. Introduction

Attention deficit hyperactivity disorder (ADHD) is identified as a neurodevelopmental condition with substantial effects on an individual's cognitive capabilities and behavioral patterns [1],[2]. Recent studies suggest that this cognitive and behavioral disorder may be significantly associated with variations in brain lateralization. Brain lateralization is also known as hemispheric dominance or brain dominance. Lateralization of the brain hemispheres refers to a functional dominance of one hemisphere over the other [3]. In cognitive processes, one is more dominant than the other. To understand cognitive strengths and weaknesses, identifying which hemisphere may be more dominant for certain functions can help individuals better understand their cognitive landscape [4].

Researchers investigating the role of brain lateralization in ADHD have illuminated potential asymmetries in neural activation patterns. For instance, in [5], researchers examined brain lateralization in individuals with ADHD by analyzing resting-state fMRI signal variability, with particular emphasis on gender differences and ADHD subtypes. The findings revealed that patients with ADHD exhibit increased rightward lateralization in the inferior frontal gyrus, precuneus, and paracentral lobule, whereas reduced rightward lateralization was observed in the insula.

Similarly, to analyze the prevalence and implications of lateralization patterns in children with ADHD compared to their typically developing peers, lateral dominance was assessed by measuring hand, foot, eye, and ear preferences, standardized questionnaires, and self-reported questionnaire data on ADHD symptoms [6]. Utilizing this dimensional approach, individuals with higher inattention symptoms demonstrated specific right hemisphere processing deficits but involved complex interactions between increased right hemispheric activity, reduced left hemispheric function, and impaired interhemispheric communication. These findings suggest that atypical lateralization, particularly in the right hemisphere, may be associated with adult inattention [7],[8]. Brain lateralization is more significant in unmedicated persons with ADHD, showing that stimulants may assist in normalizing these differences. This underlines the significance of hemisphere asymmetries as potential indicators in ADHD research [9].

Furthermore, EEG has also yielded valuable insights into the brain lateralization and brain dominance associated with healthy individuals and ADHD. The prefrontal cortex, crucial for executive functions, has been a primary area of interest [10]. EEG studies have revealed alterations in the left and right asymmetry and lateralization of neural oscillations during cognitive tasks [8],[11],[12] and resting state [13], providing evidence of atypical hemispheric engagement in individuals [14]. EEG captures the real-time dynamics of brain activity, allowing for a nuanced understanding of the neural processes contributing to ADHD symptomatology.

In our study, brain dominance is quantified using power spectral density (PSD) across EEG sub-bands. Hemispheric dominance is determined by comparing left- and right-hemispheric PSD values for each frequency band, reflecting the relative distribution of activity rather than exclusive functional control. Accordingly, we analyze differences in alpha, beta, theta, and other frequency bands between the two hemispheres. This quantification may inform future personalized interventions, such as customized neurofeedback or medication strategies [15].

Moreover, neurofeedback has been increasingly used as a treatment modality for ADHD [16]. Neurofeedback is a brain training technique that utilizes real-time EEG monitoring to help individuals learn to self-regulate their brain activity. This method is based on operant conditioning and provides visual or auditory feedback, such as a video game character moving, from brain regions associated with ADHD. By learning to control this feedback, individuals can enhance their attention, reduce impulsivity, and better regulate their arousal levels [17],[18]. The authors in [19] discussed the application and challenges of neurofeedback based on the EEG sub-bands. However, application details of neurofeedback and medication based on spectral analysis are beyond the scope of the present work.

Focusing on our research, no prior study has conducted a comprehensive statistical analysis of brain dominance across all frequency bands for control subjects, ADHD, and its subtypes, particularly considering both resting-state and working-state data. Our research explores and analyzes brainwave patterns associated with left-brain (LB) and right-brain (RB) dominance. The study examines variations in alpha, beta, theta, and other frequency bands between the left and right hemispheres of the brain, using methods such as topographical maps and statistical tests. The goal is to determine whether there is a noteworthy distinction in brainwave activity associated with left and right brain dominance, and whether these distinctions correlate with the cognitive abilities or traits of individuals with ADHD. Moreover, prior studies have incorporated EEG-based analysis, and those that do are often limited to a single dataset. In contrast, our research work performs a rigorous EEG-based lateralization analysis across five independent datasets, providing a more comprehensive and robust evaluation of hemispheric asymmetries in ADHD.

Our manuscript's overall structure consists of four sections, including this introductory section. Section 2 begins by laying out the theoretical dimensions of the research on brain lateralization and EEG. The third section concerns the methodology used for this study. The fourth section presents the research's results and findings.

## 2. Related work

This section focuses on the discussion of the literature on the spectral analysis of brain lateralization. The subsequent section provides a comprehensive literature review of both brain lateralization and spectral analysis. Current research in this area highlights significant findings and methodologies that contribute to our understanding of these concepts.

### 2.1. Spectral analysis

Prior studies have demonstrated that EEG spectral analysis is a powerful technique to analyze the frequency content of EEG signals, providing valuable insights into the underlying neural mechanisms of the brain [20]. Researchers have extensively investigated the oscillatory activity and functional connectivity by decomposing the EEG signals into different frequency bands, such as delta, theta, alpha, beta, and gamma [21] patterns within the brain.

EEG spectral analysis has been widely applied across various fields, contributing to a deeper understanding of brain activity and cognition. For instance, in cognitive neuroscience, EEG spectral analysis has been extensively used to investigate cognitive processes such as attention, perception, memory, and language [22]. By examining changes in specific frequency bands, researchers have identified neural correlates during attention and creativity performance in children [22]. Furthermore, clinical neurology studies showed that EEG spectral analysis plays a crucial role in diagnosing and monitoring various neurological disorders, including ADHD, autism, addiction, bipolar disorder, anxiety, panic disorder, post-traumatic stress disorder (PTSD), obsessive-compulsive disorder (OCD), schizophrenia [23], epilepsy [24], sleep disorders [25], and hallucinations in Alzheimer's disease [26]. Researchers have reported that abnormal spectral patterns can provide insights into the presence and progression of disorders, aiding treatment planning and patient management. EEG spectral analysis, a technique used to modulate brain activity, has also been employed in neurofeedback training. By providing real-time feedback based on specific frequency bands, individuals can learn to self-regulate their brainwaves, potentially improving attention, relaxation, and cognitive performance [27].

Likewise, prior research has shown that analyzing spectral power and functional connectivity can provide insights into the functional specialization of the left and right hemispheres of the brain [28]. Existing studies have shown that EEG signals are commonly analyzed in the frequency domain, whereas EEG rhythmic activity in certain frequency bands is typically represented as time-variant spectral power in a joint time-frequency domain, which can be estimated and characterized using a set of time-frequency analysis (TFA) methods [29]. The methods commonly used in the time-frequency domain are the fast Fourier transform (FFT) [26], the short-time Fourier transform (STFT) [15], and the wavelet transform (WT) [10],[30]. Spectral analysis fundamentally relies on the Fourier transform, from which the power spectral density (PSD) is derived to describe the distribution of signal power across frequencies [31]. Prior studies have employed several spectral estimation approaches for EEG analysis, including nonparametric periodogram methods, the Multitaper technique, parametric autoregressive model-based methods [32], and Welch's Burg method [33].

### 2.2. Brain lateralization

Related studies have described brain lateralization, defined as the specialization of cognitive functions between the brain's two hemispheres, where certain processes are more dominant in one hemisphere.

The left hemisphere is generally associated with language processing, logical reasoning, and analytical thinking, while the right hemisphere is linked with spatial awareness, creativity, and emotional processing [34]. Although functions like language and spatial perception are more specialized in one hemisphere (language in the left, spatial perception in the right), many cognitive processes require the collaboration of both hemispheres [35]. The concept of left- and right-brain dominance has been oversimplified in popular discourse, but understanding which hemisphere may be more dominant for specific functions remains essential. This understanding can reveal cognitive strengths and weaknesses, aiding in career planning and personal development [36]. Additionally, the pattern of brain lateralization has been investigated as a biomarker for specific conditions or traits, such as schizophrenia, associated with reduced left hemisphere lateralization for language [37], and autism, linked with reduced right hemisphere lateralization for spatial processing [38].

Brain lateralization has been measured through a variety of methods, including functional magnetic resonance imaging (fMRI) [5],[39], transcranial magnetic stimulation (TMS) [40],[41], and EEG. By analyzing these measures, researchers have identified deviations from typical lateralization patterns and used these deviations as a biomarker for specific conditions. In [42], authors explored how right- and left-handed individuals differ in brain activity during self-motion perception, revealing distinct patterns in the parietal and occipital regions. This suggests that handedness may affect how self-motion is processed in the brain, potentially influencing interventions for motion-related disorders. The dichotic listening test [43] also highlighted how auditory processing varies between the brain's hemispheres.

### 2.3. Brain lateralization and ADHD

Research using the Disruptive Behaviour Disorders Rating Scale (DBD) [6] suggests a link between atypical hemispheric lateralization and ADHD in children, with a possible higher incidence of left- or mixed-handedness in those with ADHD compared with typically developing children. Despite these findings, it is important to remember that brain functions often require the cooperation of both hemispheres, not just the dominant one. ADHD, a prevalent neurodevelopmental disorder, is characterized by inattention, hyperactivity, and impulsivity. Studies [7],[8] indicate that individuals with ADHD might experience disruptions in normal brain lateralization, including reduced right hemisphere activity, which could contribute to ADHD symptoms. Some research also points to more bilateral brain activity in those with ADHD, suggesting further deviations from typical lateralization patterns.

In [10], the role of frontal alpha asymmetry (FAA) in ADHD was explored. FAA refers to an EEG alpha oscillation power imbalance between the right and left frontal channels. It was observed that individuals with ADHD had lower FAA, indicating decreased activity in the left frontal cortex. Additionally, within the ADHD group, there was a notable correlation between left-lateralized FAA and improved inhibitory control, a relationship that was absent in the control group.

Research by T. Sigi Hale [13],[44] showed that children with ADHD had more significant right frontal alpha asymmetry during various conditions, implying lower activity in the left frontal cortex. Furthermore, authors in [45] found significant right-sided lateralization of gamma activity in the brains of healthy young males, emphasizing hemisphere differences in brain activity. Likewise, in a study [46] involving adults with ADHD, a significant rightward beta asymmetry was observed in the inferior parietal regions during a continuous performance task (CPT). This asymmetry was distinct and not related to linguistic skills. Among those with ADHD, a greater degree of this rightward beta asymmetry correlated with better performance on the CPT. Furthermore, unlike in control subjects, this asymmetry in ADHD individuals did not align with the usual left-biased processing in the temporal-parietal regions, key areas for advanced language functions.

While these studies contribute valuable insights into ADHD and brain lateralization, more research is needed for a comprehensive understanding. The role of brain dominance in ADHD and its treatment, particularly regarding neurofeedback, remains an area of ongoing investigation. Neurofeedback aims to train individuals to regulate their brain activity, enhancing cognitive functions like attention and self-control. Though some studies suggest ADHD may involve differences in brain activity or connectivity between hemispheres, conclusive evidence regarding the importance of brain dominance in neurofeedback for ADHD is still developing [47].

Previous studies have primarily focused on asymmetries within specific frequency bands or localized cortical regions, such as frontal alpha, parietal beta, or task-specific gamma activity. Our research aims to expand this focus by systematically analyzing all EEG sub-bands. Additionally, while existing investigations predominantly highlight frontal or parietal regions, our study employs a comprehensive hemispheric approach by examining left–right brain dynamics. Our method contributes to a more thorough understanding of hemispheric specialization and lateralization patterns in individuals with ADHD. Additionally, whereas previous studies have typically analyzed either resting-state or task-based EEG data, our research considers both conditions, allowing for a more complete assessment of brain activity patterns in ADHD.

## 3. Materials and proposed methodology

EEG helps determine whether there are significant differences in brain waves between the left and right hemispheres and whether these differences can be correlated with cognitive abilities or traits. To investigate and analyze brain waves associated with left- and right-brain dominance, we utilized EEG data in both working and resting states for this investigation. Subsequently, power spectrum analysis using the Wilcoxon rank test and Bayesian factor analysis was performed to assess brain dominance. Figure 1 shows the workflow for Brian's dominance analysis.

**Figure 1.**
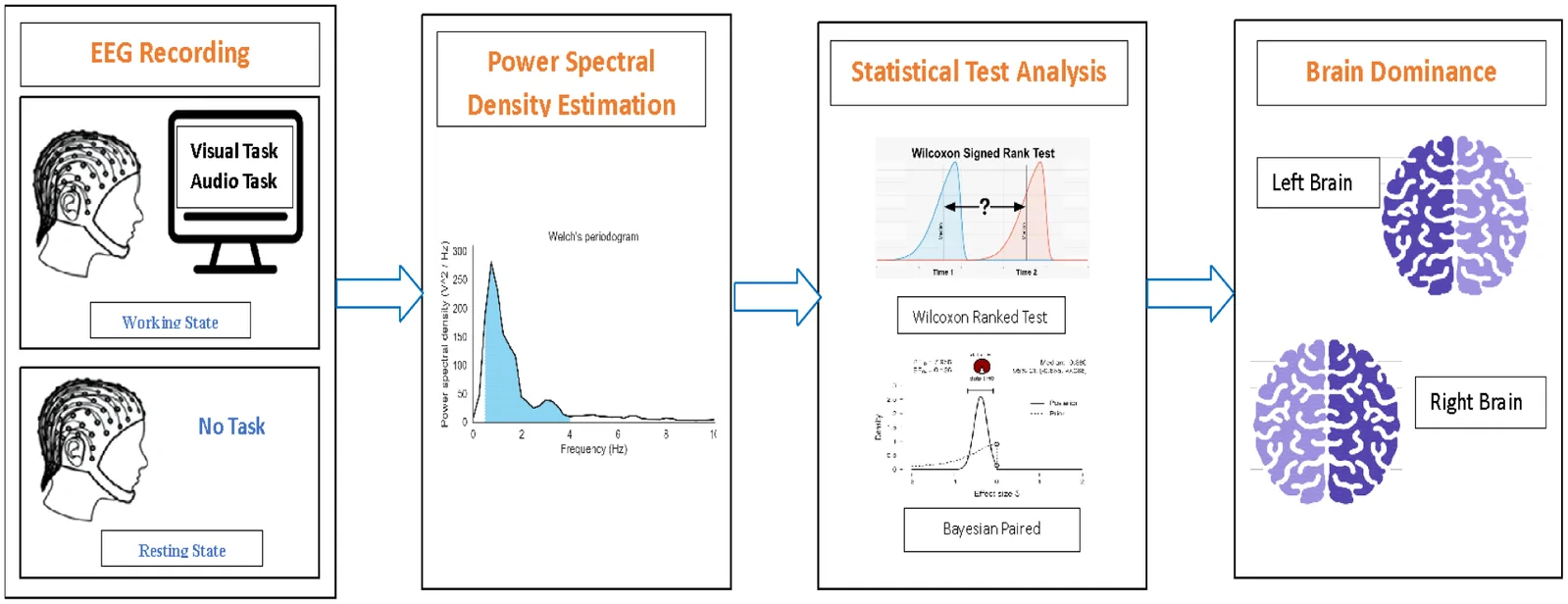
Workflow for brain dominance analysis.

We also examined the left and right brain dominance and hemisphere asymmetry sub-band analysis in inattentive, hyperactive, and combined subtypes of ADHD.

The primary objective of our research is to identify a specific sub-band frequency range that could be used as a biomarker to discriminate between different subtypes of ADHD and control individuals. We investigated whether differences in sub-band activity between subtypes can help us understand the underlying neural mechanisms that contribute to each subtype's symptoms. The results of our study could lead to a better understanding of ADHD. They may contribute to the development of more effective diagnostic tools and treatment approaches for individuals with ADHD.

### 3.1. Problem statement

We aim to study and analyze EEG brain waves in order to:

- Determine hemispheric dominance in ADHD and control groups;
- Analyze EEG sub-bands during resting state conditions, eyes closed (EC), eyes open (EO), and working state tasks;
- Study and compare EEG patterns among ADHD subtypes: inattentive, hyperactive, and combined;
- Identify potential EEG sub-band biomarkers capable of discriminating between ADHD subtypes;
- Examine the hypothesis that, in the resting state, the alpha power spectra of the left and right hemispheres are approximately equal, or that the left alpha power spectrum exceeds the right in left-brain dominant individuals.

The following subsections describe the materials, experimental design, and methodological approaches undertaken in the investigation.

### 3.2. Datasets

We considered EEG recordings at resting state (Datasets 1 and 5) and working state (Datasets 2, 3, and 4) to analyze brain dominance. Details of the datasets are given below.

Dataset 1: In this dataset [48], EEG recordings, each lasting around 90 min, were conducted on subjects at rest in a soundproof, climate-controlled room, with temperature and lighting regulated and protection against magnetic and electrical interference provided. The study involved two groups: an ADHD group of 18 individuals and a control group of 12 individuals. The EEGs were recorded at a sampling rate of 256 Hz using a Nihon Kohden Neurofax EEG-9200, both with eyes open (EO) and closed (EC). The recording utilized an analog-digital channel cap in accordance with the extended International 10/20 system, focusing on a selected set of eight channels: Fp1, Fp2, C3, C4, T3, T4, O1, and O2. Data filtering was performed in real-time with a high-pass filter (cutoff frequency: 0.05 Hz), a low-pass filter (80 Hz), and a notch filter at 50 Hz.

Dataset 2: This dataset [49], provided by Iran's National Brain Mapping Laboratory's “First EEG Data Analysis Competition with Clinical Applications”, includes brain activity recordings from two groups: 196 individuals with ADHD and 132 from a control group. The recordings were captured at a 512 Hz sampling rate using 19 channels, with filters applied to remove slow drifts below 1 Hz and high-frequency noise above 20 Hz. Participants were recorded while engaged in cognitive tasks or activities requiring mental effort. The dataset focuses explicitly on 31 children with ADHD (22 boys and 9 girls, aged 7–10) and 30 children from a normal group (25 boys and 5 girls, aged 8–11). During the recordings, these children were shown various images on a monitor, such as animals and cartoons, and were asked to count them. The key goal was to keep the children engaged in a continuous mental task during the EEG, regardless of the accuracy of their counting.

Dataset 3: This dataset [23],[50] includes EEG recordings from 20 neurotypical and 25 young adults with ADHD, recorded using 64 channels during auditory tasks. The tasks involved listening to human speech streams composed of the syllables /ba/, /da/, and /ga/. Each trial had a central “target” stream with three syllables and a “distractor” stream to the right with five syllables. Additionally, two-thirds of the trials featured an “interrupter” stream to the left, starting either 1 s (early interrupter) or 1.5 s (late interrupter) after the target. The trials were balanced across *no interrupter*, *early interrupter*, and *late interrupter* conditions. Participants received visual cues for the required attentional state, focusing on a central dot. In FOCAL attention trials, they concentrated on the target and reported the syllable sequence. In BROAD attention trials, they monitored the target and the interrupter (if present) while ignoring the right-lateralized distractors. Initially, at 2048 Hz, the recordings were downsampled to 256 Hz with filters applied to remove noise below 1 Hz and above 20 Hz. Participants performed cognitive tasks during these recordings, requiring mental effort.

Dataset 4: This dataset [51] is a collection of recordings of brain activity in individuals with different subtypes of ADHD. The inattentive subtype comprises 21 individuals, while the combined subtype includes 12 individuals. Only one individual with the hyperactive subtype is included in this dataset. The recordings were taken using 21 channels at a sampling rate of 500 Hz. A filtering process was applied to the recordings to filter out noise and artifacts. However, the details of the filtering process are not mentioned in the available information. The recordings were taken during a working state that involved participants performing cognitive tasks or engaging in activities that required mental effort. Specifically, the NoGo task was used, which requires individuals to withhold a response when presented with a particular stimulus. This dataset can be used to gain insights into the neural activity of individuals with different ADHD subtypes during the NoGo task. It may provide valuable information for future research in this area.

Dataset 5: This dataset [52] consists of recordings of brain activity in individuals with different subtypes of ADHD. The inattentive subtype comprises 48 individuals, while the combined subtype includes 45 individuals. Only two individuals with the hyperactive subtype are included in this dataset. Participants were in a resting state during the recording process and were instructed to keep their eyes open. The recordings were taken using 21 channels at a high sampling rate of 2000 Hz/channel, later downsampled to 250 Hz. The recordings were filtered to remove unwanted noise and artifacts using a notch filter with a 55–65 Hz frequency range, a low-pass filter with a 0.3 Hz cutoff, and a high-pass filter with a 30 Hz cutoff.

Table 1 summarizes the characteristics of the EEG datasets used in our study, including the number of subjects, age ranges, recording channels, sampling rates, recording states, durations, and medication statuses. It includes resting-state and working-state EEG recordings from ADHD and control participants across various age groups, along with variations in recording conditions and medication statuses.

### 3.3. Spectral estimation

The key objective of our study is to observe and characterize hemispheric brain dominance in ADHD, in ADHD subtypes, and in controls using EEG spectral features. Previous studies [28],[53] showed that the dominant brain hemisphere of a person can be identified by comparing PSD values between the two hemispheres of the brain. PSD is a widely used method for quantifying and examining EEG signals. Researchers can gain insights into the signal's frequency characteristics and understand how power is distributed across frequencies. This analysis involves deriving several parameters from the power spectrum, including total power, spectral-band power, median frequency, and spectral-edge frequency.

A power spectrum describes the energy distribution of a time series in the frequency domain. PSD estimation can be done using parametric (autoregressive model) and nonparametric (Multitaper method, Welch's method, and Burg method) methods. We calculate PSD using the nonparametric Welch's method. The key benefit of nonparametric methods is their robustness, as estimated PSDs do not contain spurious frequency peaks. In contrast, parametric methods do not use data windowing. Parametric methods assume that a signal fits a particular model. The estimated PSDs may contain spurious frequency peaks if the assumed model is wrong. If the assumed model is correct, PSDs estimated with parametric methods are less biased and possess a lower variance than those estimated with nonparametric methods. However, the magnitudes of PSDs estimated with parametric methods are usually incorrect [32]. Band power in our work is therefore calculated from the total PSD using the Welch method; therefore, the obtained values include both oscillatory and aperiodic components of the EEG spectrum.

**Table 1.** Summary of the dataset.

Dataset	Number of subjects/age	Number of channels/sampling rate	Recording state with recording length	Medication state
Dataset 1	ADHD-18 Control-12 Age: 6–9	8/256 HZ	Resting state: EO, EC 3–5 min	Not specified
Dataset 2	ADHD-31 Control-30 Age: 7–11	19/512Hz	Working-visual stimuli 4 min	Children had been taking Ritalin for up to 6 months.
Dataset 3	ADHD-25 (Inattentive-12, Combined-10, Hyperactive-3) Control-20 Age: 21.4 +/−2.7	64/256 Hz	Working-audio stimuli Not specified	Randomized to on- or off-stimulant medication at day 1; subset (N = 14) repeated in opposite state on day 2, but due to practice effects, only day 1 data were analyzed.
Dataset 4	ADHD-34 (Inattentive-21 Combined-12 Hyperactive-1) Control-25 Age: 9–16	21/500Hz	Working-visual stimuli 19 min	Eleven patients took ADHD medication but refrained from that for at least 24 h before the experiment.
Dataset 5	Inattentive-48 Combined-45 Hyperactive-2 Age: 18–21	21/250 Hz	Resting state: EO Not specified	Not specified

The calculation of Welch's is executed in Python using the *scipy.signal.welch* library. Welch's method estimates the power spectrum by dividing the signal into overlapping segments, computing the periodogram for each segment, and averaging the results. Let *x*(*n*) denote the discrete time-domain signal. The *mth* windowed segment of the signal is defined as Eq 1:

$$x_{m}(n)≜w(n)x(n+mR),n=0,1\ldots M-1,m=0,1,\ldots .K-1$$ (1)

where *w*(*n*) is the window function, *M* represents the window length, *R* denotes the hop size between successive segments, and *K* is the total number of segments.

The periodogram of the *m^th^* segment is computed using the Fast Fourier Transform (FFT) as shown in Eq 2:

$$P_{xm,M}\left(w_{k\;}\right)=\frac{1}{M\;}{\left|FFT_{N,k}\left(x_{m}\right)\right|}^{2}≜\frac{1}{M}{\left|\overset{N-1}{\underset{n=0}{\sum }}x_{m}\left(n\right)e^{-\frac{j2\pi nk}{N}}\right|}^{2}$$ (2)

where *N* denotes the FFT length, and *k* represents the frequency bin index.

The Welch estimate of the power spectral density is given by Eq 3:

$$s_{x}^{W}\left(w_{k}\right)≜\;\frac{1}{K}\overset{k-1}{\underset{m=0}{\sum }}P_{x_{m,M}}(w_{k})$$ (3)

In other words, it is simply the average of periodograms over time. When *w*(*n*) is the rectangular window, the periodograms are formed from non-overlapping successive data blocks.

Figure 2 shows PSD examples for the left and right channels in the EC state.

**Figure 2.**
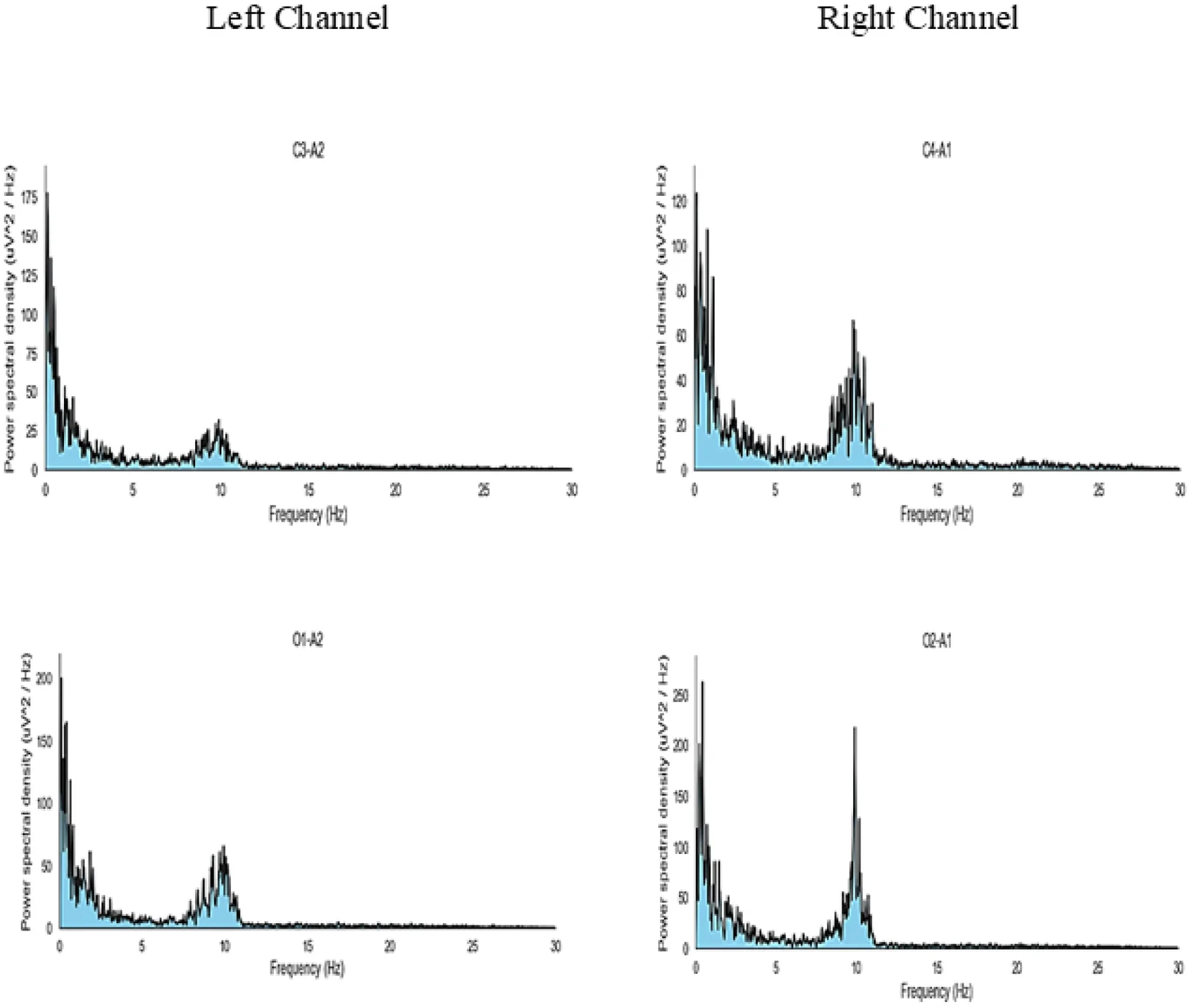
Average PSD for an ADHD subject in the EC state at age 9.

Our study employed PSD values for various analytical purposes. The subsequent subsection describes the specifics of these analyses.

### 3.4. Asymmetric index

Average PSD was computed by considering the frequency band range from 1 to 30 Hz for both the ADHD and control groups. To assess differences in average PSD across the hemispheres, we calculated the asymmetry between the right and left hemispheres (A_PSD_RL) for each participant within the ADHD and control individuals. The Asymmetric Index was determined using the formula given in Eq 4.

$$Asymmetric\;Index:\Delta RL=R_{i}-L_{i}\;for\;i=1,2,3\ldots \ldots N$$ (4)

where N is the total number of subjects.

The positive value of the index signifies that, on average, the power-spectrum values of the right hemisphere were higher than those of the left, and vice versa.

### 3.5. Brain dominance analysis for the ADHD and control groups

In our research, we analyzed the Asymmetric Index during resting and working states of EEGs in ADHD and control subjects. As detailed in Table 2, the investigation revealed that ADHD subjects (80% in dataset 2 and 58% in dataset 3) exhibited a positive index during working-state EEG, suggesting a predilection for higher average power spectral density (AVG PSD) values in the right hemisphere. In contrast, the control group showed no significant difference in PSD between the left and right hemispheres.

Furthermore, in the resting-state dataset (Dataset1), particularly in the EC state, 59% of ADHD subjects presented a high PSD value in the left brain (LB). Conversely, 55% of control subjects found high PSD in the right brain (RB). In the EO condition, a significant percentage (ranging from 80% to 90%) of both ADHD and control subjects exhibited higher PSD values in the right hemisphere.

These findings suggest several key insights. First, average PSD values across the brain's hemispheres will help determine the predominant cerebral hemisphere in an individual. Second, ADHD subjects tend to have higher average PSD in the right hemisphere during active states, but this shifts to the left hemisphere during resting states. Moreover, we also found that in 34% of cases, there were notable variations in PSD between the left and right hemispheres during EC and EO states. This tendency was particularly evident in children aged 6 and 9.

**Table 2.** Left and right brain dominance analysis based on AVG_PSD for the ADHD and control groups.

Dataset	Left/right brain	Control (%)	ADHD (%)
Dataset 3 (working state)	Left brain	50	20
	Right brain	50	80
Dataset 2 (working state)	Left brain	66	42
	Right brain	34	58
Dataset 1 (resting-state) EC	Left brain	45	59
	Right brain	55	41
Dataset 1 (resting-state) EO	Left brain	20	01
	Right brain	80	99

#### 3.5.1. Hypothesis examination

After analyzing the AVG PSD, we computed the PSD values for alpha, beta, theta, and delta brain waves. These brain waves were studied and analyzed to investigate the following hypothesis:

*Hypothesis 1*: In the resting state, it is expected that the alpha power spectra for the left and right hemispheres are nearly equal or that the left alpha power spectrum is higher than the right if the subject is left-brain dominant.

In investigating Hypothesis 1, our findings indicated an asymmetry (not equal) in alpha power between the left and right cerebral hemispheres. As illustrated in Figure 3, among the 18 participants diagnosed with ADHD, 11 exhibited a higher alpha PSD in the left hemisphere. Similarly, in the control group of 12 subjects, 7 demonstrated elevated alpha PSD in the left hemisphere.

**Figure 3.**
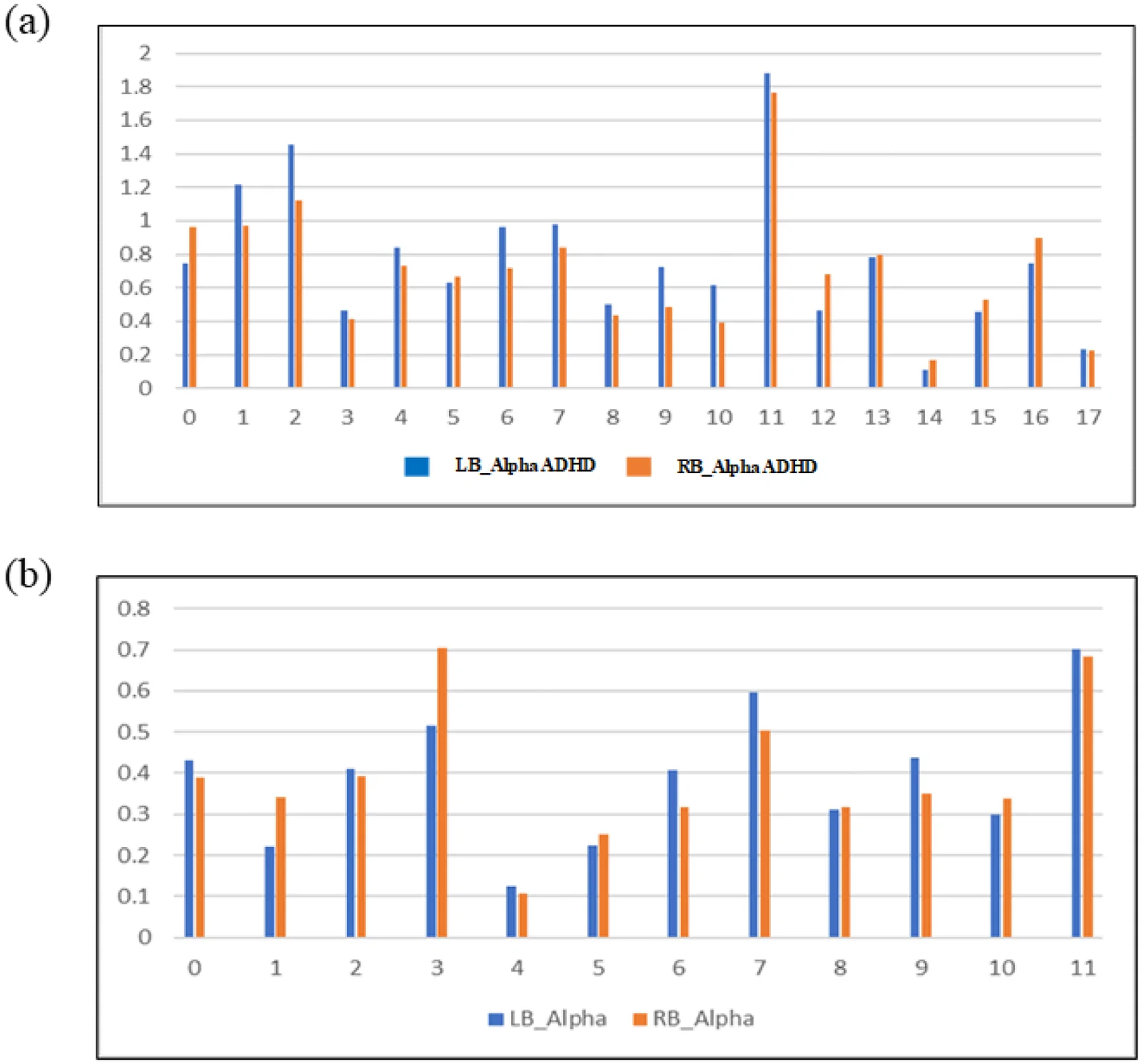
Analysis of alpha wave power (a) in EC state for ADHD and (b) in EC state for control, x-axis subjects, y-axis alpha wave power.

#### 3.5.2. Functional hemisphere sub-band asymmetry for brain dominance for the ADHD and control groups

Our research involved examining and analyzing brain wave patterns in ADHD and control subjects to determine left and right brain dominance and identify potential biomarker sub-bands. Using topographic visualization and the Wilcoxon rank-sum test, we examined the asymmetry of hemispheric brain waves to assess brain dominance in the ADHD and control groups. This study also aimed to analyze differences in energy distribution across brain regions and pinpoint specific areas with increased power.

*Topographical analysis*: Topographical maps are primarily used for visualization, to check the distribution of band power across the brain. This method helps identify significant spatial differences between the ADHD and control groups. The topographical view used a color scale, ranging from red to blue, with red representing maximum power and blue indicating minimum power, as illustrated in Figure 4. In Figure 4, we show only a subset of samples from Dataset 3 for ADHD (Figure 4a,b) and control groups (Figure 4c,d). Dataset 3, with a larger number of channels (64), can also be extended to other datasets.

The biggest differentiator between ADHD and control groups is the delta: ADHD subjects show whole-scalp high delta, while control subjects show limited frontal delta. Theta is present in both groups, but ADHD shows a larger spatial spread. Alpha and beta do not clearly separate the two groups at the individual level.

Further analysis, as shown in Figure 5, compares the cumulative power across all ADHD and control subjects. ADHD is characterized by a pronounced increase in delta activity, particularly in the left frontal region, indicative of elevated slow-wave power. In contrast, the control group exhibits a more fragmented distribution of delta activity, lacking a clear spatial organization. While theta activity shows a mild elevation in the frontal-central region for individuals with ADHD, it remains consistently low among the control group. Both alpha and beta frequencies show comparably low levels and lack distinctive features across both groups. These observations are based on the topographical analysis of Dataset 3, presented as a representative sample. Within this dataset, the group-average maps suggest that ADHD is associated with delta dominance localized in the left frontal region, along with moderate theta elevation, while the control group does not demonstrate a consistent spatial power organization across the examined frequency bands.

**Figure 4.**
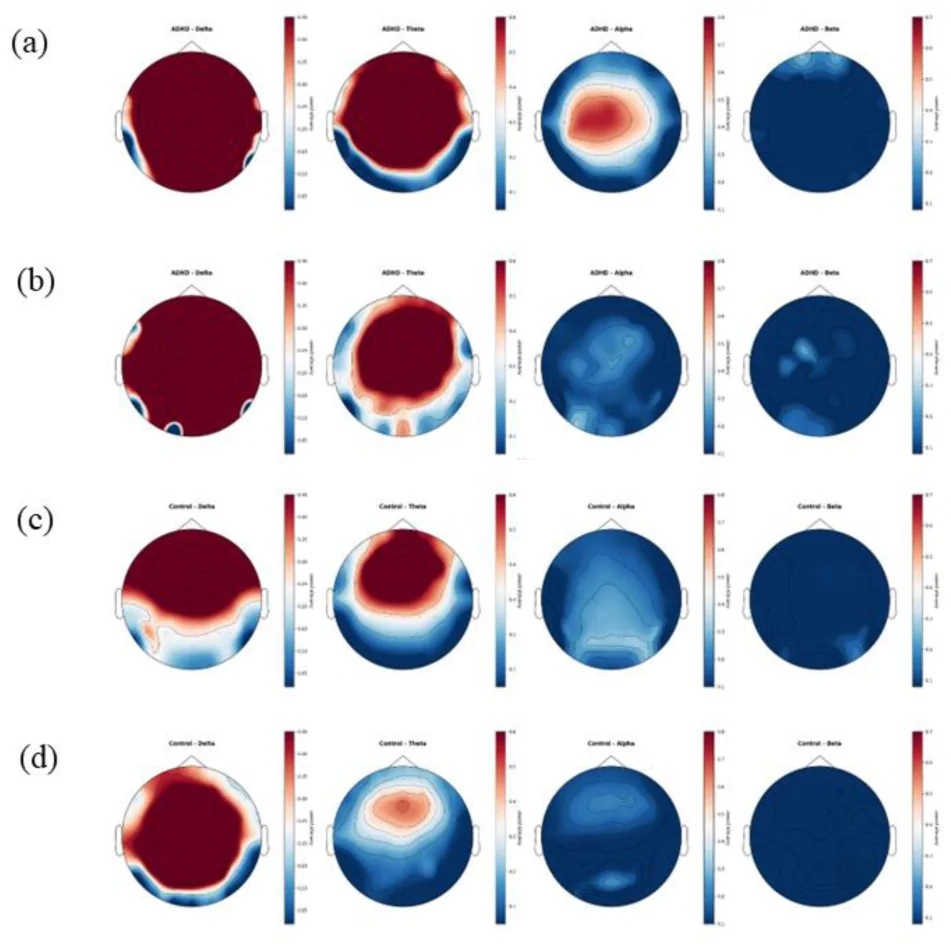
Samples of topographical view of an ADHD subject (a, b) and a control subject (c, d) from Dataset 3.

**Figure 5.**
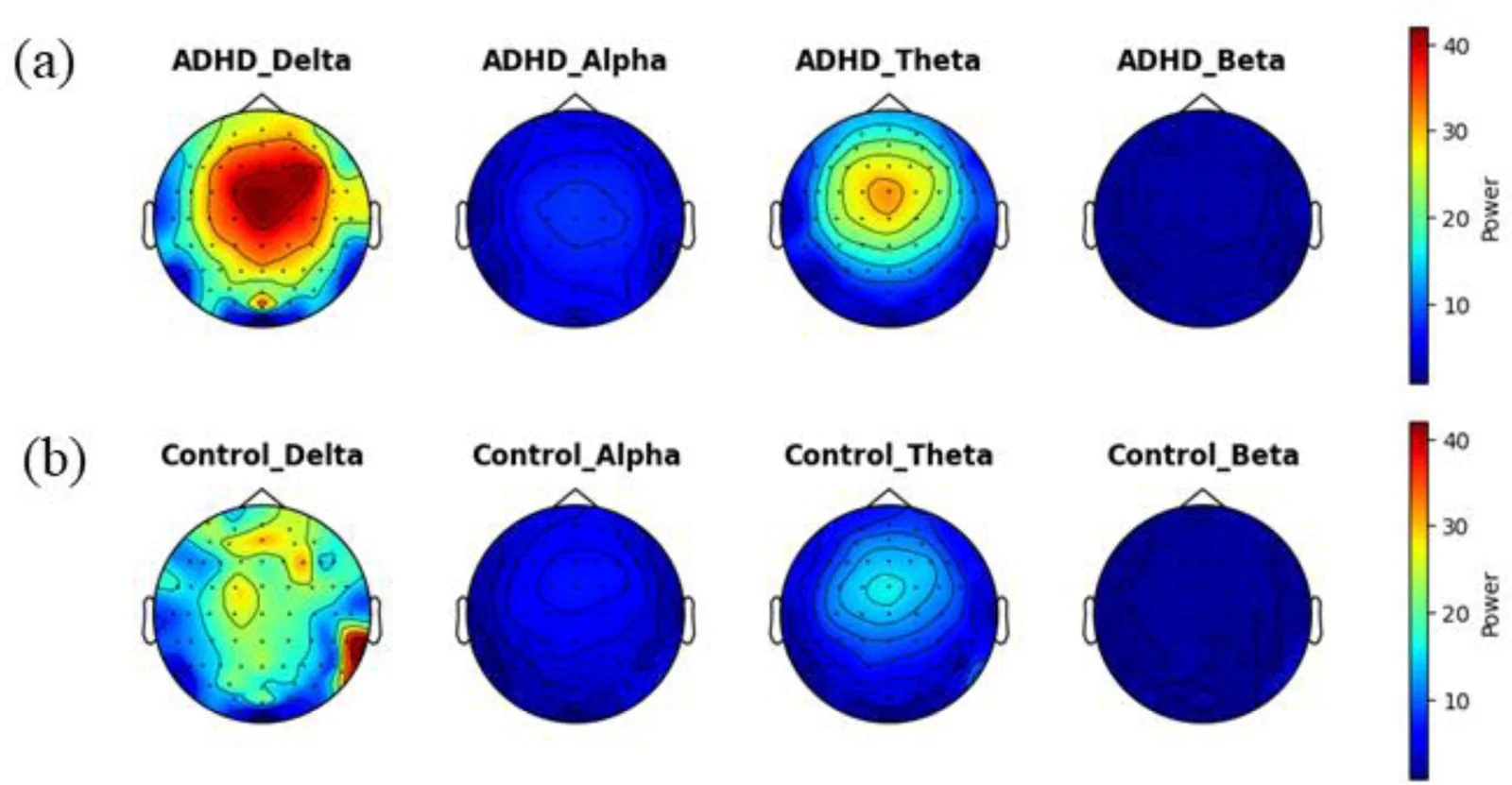
Topographical view of the cumulative power of all brain waves: (a) cumulative power for the ADHD group and (b) cumulative power for the control group for Dataset 3.

### 3.6. Statistical analysis to validate topographic analysis

We used the Wilcoxon signed-rank paired test to corroborate the topographical view findings. In this nonparametric method, we assume the following hypotheses:

*Null hypothesis H_0_: PSD of LB = PSD of RB*

*Alternative hypothesis H_1_: PSD of LB < PSD of RB*

The alternative hypothesis specifies that Measure 1 is less than Measure 2 for all tests. For example, AVG_LB is less than AVG_RB.

For computing the PSD of LB, all left-side and right-side channels are considered for RB.

The Wilcoxon test was performed in the JASP open-source statistics program to focus on pairwise comparisons such as AVG_LB vs. AVG_RB, Delta_LB vs. Delta_RB, Theta_LB vs. Theta_RB, and Beta_LB vs. Beta_RB. We adopted a 95% confidence interval for our hypothesis testing.

The null hypothesis has been defined, with one-sided alternatives specified where appropriate, and the results have been interpreted cautiously, particularly for marginal p-values. This method was employed to investigate hemispheric asymmetry in sub-bands of EEG data for both ADHD and control subjects, using resting-state and active-state EEG recordings.

### 3.7. Resting state analysis of sub-band asymmetry for the ADHD and control groups

In our examination of the resting-state EEG data from the EC and EO conditions of Dataset 1, no notable differences were observed in the PSD values, as observed by the P-value > 0.05 between the left brain (LB) and right brain (RB) in ADHD subjects (refer to Tables 3 and 5, respectively). In contrast, the control group showed a significant variance in the beta wave, with a p-value of less than 0.05, in the EC state, as detailed in Table 4; significant differences were observed in the delta and beta waves of LB and RB, in the EO state, as shown in Table 6.

**Table 3.** Paired samples Wilcoxon rank test result for the EC state of the ADHD group of Dataset 1.

Measure 1	Measure 2	z	P
AVG_LB	AVG_RB	1.285	0.902
Delta_LB	Delta_RB	0.719	0.766
Theta_LB	Theta_RB	0.457	0.680
Alpha_LB	Alpha_RB	1.938	0.976
Beta_LB	Beta_RB	−0.893	0.196

**Table 4.** Paired samples Wilcoxon rank test result for the EC state of the control group of Dataset 1.

Measure 1	Measure 2	z	P
AVG_LB	AVG_RB	−0.7845	0.2349
Delta_LB	Delta_RB	−0.6276	0.2847
Theta_LB	Theta_RB	0.0784	0.5452
Alpha_LB	Alpha_RB	−0.4707	0.3386
Beta_LB	Beta_RB	−1.7258	0.0461

**Table 5.** Paired samples Wilcoxon rank test result for the EO state of the ADHD group of Dataset 1.

Measure 1	Measure 2	z	P
AVG_LB	AVG_RB	0.8928	0.8154
Delta_LB	Delta_RB	1.1105	0.8677
Theta_LB	Theta_RB	0.8057	0.7914
Alpha_LB	Alpha_RB	−0.6750	0.2613
Beta_LB	Beta_RB	−1.3283	0.0982

**Table 6.** Paired samples Wilcoxon rank test result for the EO state of the control group of Dataset 1.

Measure 1	Measure 2	z	P
AVG_LB	AVG_RB	−1.883	0.032
Delta_LB	Delta_RB	−1.883	0.032
Theta_LB	Theta_RB	0.706	0.765
Alpha_LB	Alpha_RB	−1.412	0.088
Beta_LB	Beta_RB	−1.647	0.055

### 3.8. Working state analysis of sub-band asymmetry for the ADHD and control groups

Two distinct datasets were used to analyze the working state EEG, each representing different working conditions: Dataset 3, involving auditory stimuli, and Dataset 2, incorporating visual stimuli. Analysis of the control group sub-band in both datasets using the Wilcoxon test showed no significant differences in LB and RB (refer to Tables 7 and 8). This suggests that in ADHD subjects, the PSD of delta waves were consistently higher on the right-brain side across both Dataset 3 (Table 9) and 2 (Table 10). In addition, beta PSD was significantly higher on the right side in Dataset 2. No statistically significant differences were observed for theta or alpha waves; however, theta showed a non-significant trend toward higher PSD on the left side in Dataset 3, while alpha showed a non-significant trend toward higher PSD on the left side in Dataset 2 and on the right side in Dataset 3.

Consequently, as the majority sub-band PSD was found to be higher in LB for ADHD, we expanded our analysis to identify where the PSD increased in a specific region inside the left hemisphere. Comparing the left frontal and occipital regions, the results, as shown in Table 11, indicate that all bands demonstrated higher PSD in the frontal region compared to the occipital.

**Table 7.** Paired-samples Wilcoxon rank test result for the working state of the control group of Dataset 3.

Measure 1	Measure 2	z	P
AVG_LB	AVG_RB	−0.5171	0.3161
Delta_LB	Delta_RB	−0.4654	0.3343
Theta_LB	Theta_RB	−0.4137	0.3529
Alpha_LB	Alpha_RB	−1.0342	0.1613
Beta_LB	Beta_RB	−1.0859	0.1489

**Table 8.** Paired-samples Wilcoxon rank test result for the working state of the control group of Dataset 2.

Measure 1	Measure 2	z	P
AVG_LB	AVG_RB	−2.9209	0.9983
Delta_LB	Delta_RB	−0.0909	0.4643
Theta_LB	Theta_RB	0.4656	0.6797
Alpha_LB	Alpha_RB	−0.1136	0.4552
Beta_LB	Beta_RB	−1.2424	0.1073

**Table 9.** Paired-samples Wilcoxon rank test result for the working state of the ADHD group of Dataset 3.

Measure 1	Measure 2	z	P
AVG_LB	AVG_RB	−2.4620	0.0062
Delta_LB	Delta_RB	−2.9463	0.0011
Theta_LB	Theta_RB	1.5202	0.9367
Alpha_LB	Alpha_RB	−1.3857	0.0865
Beta_LB	Beta_RB	−0.9552	0.1763

**Table 10.** Paired-samples Wilcoxon rank test result for the working state of the ADHD group of Dataset 2.

Measure 1	Measure 2	z	P
AVG_LB	AVG_RB	−2.1656	0.0152
Delta_LB	Delta_RB	−3.7666	<0.001
Theta_LB	Theta_RB	−0.0013	0.4997
Alpha_LB	Alpha_RB	2.4700	0.9933
Beta_LB	Beta_RB	−5.5008	<0.001

**Table 11.** Paired-samples Wilcoxon rank test result for frontal LB vs. occipital LB.

Measure 1	Measure 2	z	P
Fr_AVG_LB	OCI_AVG_RB	4.3724	<0.001
Fr_Delta_LB	OCI_Delta_RB	4.0764	<0.001
Fr_Theta_LB	OCI_Theta_RB	4.3724	<0.001
Fr_Alpha_LB	OCI_Alpha_RB	2.8118	0.0019
Fr_Beta_LB	OCI_Beta_RB	4.2647	<0.001

### 3.9. Brain dominance analysis for ADHD subtypes

By employing the methodology used in the ADHD and control group analysis, we computed the Asymmetry Index for subjects with combined and inattentive ADHD. This index showed a positive value, indicating higher PSD in the right brain, observed in 65%–78% of both inattentive and combined subjects across both working conditions (Dataset 3 and Dataset 4). This suggests a tendency to higher right-brain PSD in active tasks. In the resting state, 54% right-brain dominance was observed for the inattentive group, and 54% left-brain dominance was observed for the combined group (Table 12).

**Table 12.** Left and right brain dominance analysis based on AVG_PSD for the ADHD subtype group.

Dataset	Left/right brain	Inattentive (%)	Combined (%)
Dataset 5 (resting state)	Left brain	47	54
	Right brain	54	47
Dataset 3 (working state)	Left brain	35	29
	Right brain	65	71
Dataset 4 (working state)	Left brain	33	21
	Right brain	66	78

#### 3.9.1. Functional hemisphere sub-band asymmetry for ADHD subtype

Sub-band asymmetry across hemispheres has been linked to core ADHD symptoms, such as inattention and impulsivity. Evidence suggests that these asymmetry patterns differ between subtypes, making them a potential marker for distinguishing inattentive and combined individuals. Our study investigated brain wave patterns in subjects with ADHD subtypes utilizing topographic visualization and the Wilcoxon statistical test. This helps to examine hemispheric brain wave asymmetry and determine brain dominance in inattentive, hyperactive, and combined groups.

*Topographical analysis of sub-band asymmetry for the hyperactive subtype*. Due to the limited number of samples of hyperactive subjects, statistical analysis is not possible; as such, for hyperactive subjects, only topographical analysis was performed. In the sub-band analysis, we observed distinct patterns for all three subjects; the power of all sub-bands is primarily distributed in the partial lobe (e.g., Figure 6a,c). Likewise, the power of delta and theta is distributed in the central and frontal lobes (e.g., Figure 6a,c). Since these observations are based on only a few samples, we cannot justify our claims for this instance with certainty.

**Figure 6.**
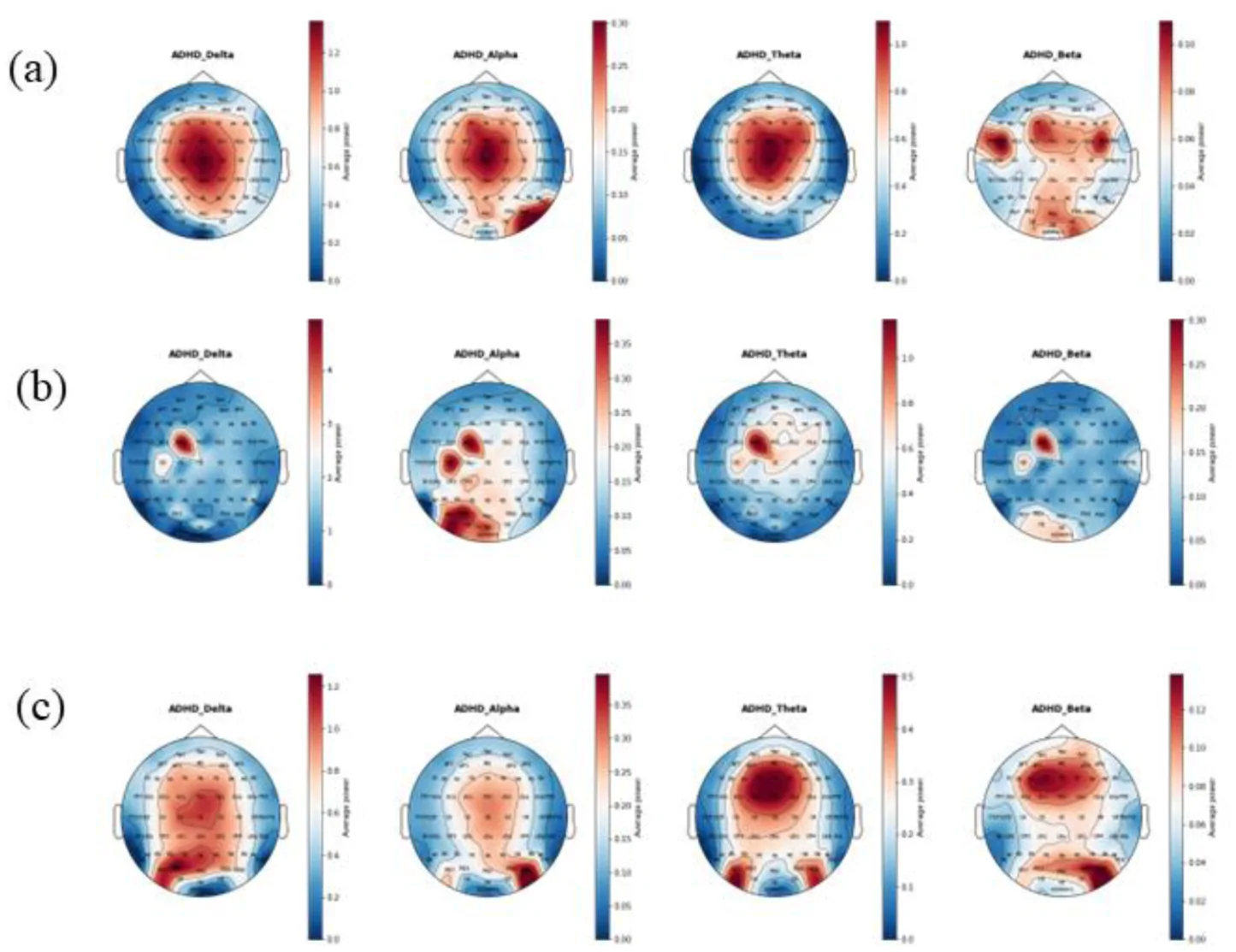
Topographical view for the hyperactive subtype: (a) view of Subject 1; (b) view of Subject 2; and (c) view of Subject 3 of Dataset 3.

### 3.10. Statistical analysis for combined and inattentive subtypes

Furthermore, we investigated sub-band asymmetries within the combined and inattentive ADHD subtypes, analyzing resting- and working-state EEG data separately for left- and right-hemisphere assessments.

To validate the results of the Wilcoxon signed-rank test, we also used Bayes Factor analysis as a probabilistic alternative to traditional p-values. The Bayesian paired-sample t-test provides a quantitative assessment of Bayes factors, which indicate the relative support for the null hypothesis (H0) versus the alternative hypothesis (H1). Specifically, the Bayes factor, denoted as BF_-0_, is calculated to facilitate this comparison, as follows:

BF_-0_ = P (Data | H_1_) / P (Data | H_0_)

where P (Data | H_1_) is the probability of the data provided that H_1_ is true, and P (Data | H_0_) is the probability provided that H_0_ is true.

In our case, our hypotheses are:

*Null hypothesis* H_0_: PSD of LB (left brain) ≥ PSD of RB (right brain)

*Alternative hypothesis* H_1_: PSD of LB < PSD of RB

In this context, we can interpret BF_-0_ = 8 as indicating 8 times stronger evidence for the claim that the PSD of the LB is less than that of RB.

Bayesian analyses were conducted in JASP using default prior settings. A Cauchy prior centered at zero with a scale parameter of 0.707 was used for the effect size, which is the standard prior recommended for Bayesian hypothesis testing.

### 3.11. Resting state analysis of sub-band asymmetry for combined and inattentive subtypes

Significant findings were observed in the beta wave in the combined subtype during the EO state. This is shown in Table 13. Similar findings were also obtained from the Bayes factor. As shown in supplementary Figure 1, BF_-0_ = 7.956 quantifies the evidence for the alternative hypothesis regarding the beta wave, indicating that the beta PSD is lower in LB.

**Table 13.** Paired-samples Wilcoxon rank test result for the EO state of the combined subtype of Dataset 5.

Measure 1	Measure 2	z	P
Delta_LB	Delta_RB	0.5700	0.7158
Theta_LB	Theta_RB	3.3693	0.9998
Alpha_LB	Alpha_RB	0.1298	0.5533
Beta_LB	Beta_RB	-2.2744	0.0111

**Table 14.** Paired-samples Wilcoxon rank test result for the EO state of the inattentive subtype of Dataset 5.

Measure 1	Measure 2	z	P
Delta_LB	Delta_RB	-0.8103	0.2121
Theta_LB	Theta_RB	1.0359	0.8496
Alpha_LB	Alpha_RB	-0.0821	0.4696
Beta_LB	Beta_RB	0.3282	0.6294

In the case of the inattentive subtype, our analysis revealed no significant findings among the sub-bands during the EO resting state (Table 14). Bayes factor analysis produced comparable results, indicating that the BF_-0_ is lower, providing stronger evidence in support of the null hypothesis. This means that, in the inattentive condition, all sub-band values were higher in the LB, as shown in Supplementary Figure 2.

### 3.12. Working state analysis of sub-band asymmetry for combined and inattentive

In the working state, for combined subjects using Dataset 3, significant results were observed in the delta wave, as presented in Table 15. Similar results were obtained with the Bayes factor, yielding a BF_-0_ value of 2.721 for the delta wave, indicating evidence for the alternative hypothesis, as shown in Supplementary Figure 3. Moreover, Dataset 4 showed significant asymmetry in both delta and alpha waves, as shown in Table 16. While the Bayes factor for these waves remained consistent, it also provided some evidence supporting the alternative hypothesis in the theta waves, yielding a Bayes factor of BF_-0_ = 2.44 for Dataset 4, as shown in Supplementary Figure 4.

**Table 15.** Paired-samples Wilcoxon rank test result for the working state of the combined subtype of Dataset 3.

Measure 1	Measure 2	z	P
AVG_LB	AVG_RB	−1.7752	0.0398
Delta_LB	Delta_RB	−1.9645	0.0253
Theta_LB	Theta_RB	2.6273	0.9972
Alpha_LB	Alpha_RB	−0.2130	0.4268
Beta_LB	Beta_RB	−1.7752	0.0398

**Table 16.** Paired-samples Wilcoxon rank test result for the working state of the combined subtype of Dataset 4.

Measure 1	Measure 2	z	P
AVG_LB	AVG_RB	−2.2773	0.0120
Delta_LB	Delta_RB	−2.2071	0.0132
Theta_LB	Theta_RB	−1.5815	0.0597
Alpha_LB	Alpha_RB	−1.8943	0.02998
Beta_LB	Beta_RB	−0.9211	0.1869

For inattentive subjects, notable findings were identified across both datasets. In Dataset 3, only the delta wave showed a significant difference, as presented in Table 17. The Bayes factor result remains consistent, as shown in Supplementary Figure 5.

In contrast, Dataset 4 showed significant results for both beta and alpha waves, as detailed in Table 18, providing evidence for the alternative hypothesis, with BF_-0_ values of 3.97 for beta and 9.654 for alpha, as shown in Supplementary Figure 6.

**Table 17.** Paired-samples Wilcoxon rank test result for the working state of the inattentive subtype of Dataset 3.

Measure 1	Measure 2	z	P
AVG_LB	AVG_RB	−1.3491	0.0950
Delta_LB	Delta_RB	−1.7752	0.0398
Theta_LB	Theta_RB	0.9231	0.8236
Alpha_LB	Alpha_RB	−1.1598	0.1317
Beta_LB	Beta_RB	0.2130	0.5912

**Table 18.** Paired-samples Wilcoxon rank test result for the working state of the inattentive subtype of Dataset 4.

Measure 1	Measure 2	z	P
AVG_LB	AVG_RB	−1.3336	0.1018
Delta_LB	Delta_RB	0.4707	0.6890
Theta_LB	Theta_RB	−0.4707	0.3386
Alpha_LB	Alpha_RB	−2.2749	0.0105
Beta_LB	Beta_RB	−1.8827	0.0320

## 4. Discussion

In our brain wave analysis for ADHD and control dominance, our findings on theta, alpha, and beta band asymmetries are broadly consistent with prior literature on ADHD. Theta activity was observed to be more prominent in the left hemisphere during active working conditions, while greater values were noted in the right hemisphere during resting states. This corresponds with findings suggesting that excessive theta in the left hemisphere may lead to disorganization, whereas heightened theta in the right hemisphere has been associated with impulsivity. These results are consistent with previous literature reporting comparable hemispheric asymmetries in individuals with ADHD [11].

Similarly, beta asymmetry demonstrated a more pronounced rightward dominance while at rest, consistent with previous research [46], indicating that ADHD reveals increased rightward beta asymmetry in the inferior parietal area during cognitive activities, which is often linked to increased anxiety. Alpha activity also demonstrated asymmetry [10],[13], with left-hemispheric dominance during working states and rightward dominance during rest. Given that alpha reflects reduced cortical activation, this pattern may indicate altered inhibitory control processes in ADHD.

Additionally, our study extends the analysis to ADHD subtypes, including inattentive and combined subtypes. In the combined group, power distribution varied by state, with rightward dominance during working conditions and a shift toward the left hemisphere at rest, alongside frequency-specific asymmetries (rightward beta vs. leftward theta/alpha). This variability suggests a more dynamic interplay between hemispheres in the combined subtype. The inattentive group showed consistent left-hemisphere dominance across all sub-bands, regardless of state, as supported by statistical tests. Such stability may represent a potential neurophysiological marker of the inattentive subtype, underscoring the importance of lateralization patterns in characterizing heterogeneity in ADHD.

## 5. Conclusions

In conclusion, the extensive study on power spectrum density (PSD) in ADHD and control subjects reveals notable insights into the asymmetry of the brain hemisphere and its potential role in identifying ADHD and its subtypes. The findings, aided by the Asymmetric Index, suggest a differential PSD pattern in ADHD subjects, with a higher average PSD in the right hemisphere during working states and in the left hemisphere during resting states. This contrasts with control subjects, who show non-significant left–right PSD difference. Examining specific brain waves, such as alpha, beta, theta, and delta, further highlights their asymmetries. In particular, theta and alpha waves are more dominant in the left hemisphere, irrespective of the task state. Theta waves are notably prominent in the frontal region, indicating their potential as biomarkers for ADHD.

In the literature, it is shown that individuals with right-hemispheric dominance often exhibit a natural tendency toward creative and imaginative thinking [54],[55]. In our work, the increased PSD values observed in the right hemisphere during the working state of participants with ADHD suggest a notable right-brain dominance. This observation would indicate a potential propensity for creativity-related cognitive processing among ADHD individuals. By understanding these patterns, educators and therapists can create targeted learning strategies that improve focus and cognitive skills while nurturing creativity. This approach can transform the brain activity related to ADHD into a valuable asset for learning and personal development [56],[57].

Statistical analyses and topographical views support all our findings, providing specific neurobiological insights into ADHD and its subtypes and improving diagnostic precision in addition to behavioral evaluations. Our findings could also aid the development of specialized interventions, including customized neurofeedback and medication strategies for individual brain activity patterns. Our analysis provides opportunities to use EEG to track treatment progress and could help in the early detection of individuals at risk. This research adds medical and clinical merit by demonstrating the connection between the electrophysiological profiles of ADHD subjects and their clinical observations.

## Use of AI tools declaration

The authors declare they have not used Artificial Intelligence (AI) tools in the creation of this article.


