# Supplementary material for: A comprehensive investigation into EEG spectral analysis for ADHD brain dominance
Source: AIMS Neurosci. 2026 Jul 8;13(3):389–414. doi: 10.3934/Neuroscience.2026017 (PMC13658093; doi:10.3934/Neuroscience.2026017)
Supplement: Supplementary file 1 [file neurosci-13-03-017-s001.pdf]

Neuroscience

AIMS Neuroscience, 13(3): 389–414.
DOI: 10.3934/Neuroscience.2026017
Received: 31 December 2025
Revised: 18 March 2026
Accepted: 31 March 2026
Published: 08 July 2026

https://www.aimspress.com/journal/neuroscience

*Research article*

# A comprehensive investigation into EEG spectral analysis for ADHD brain dominance

**Vandana Joshi*** **and Nirali Nanavati**

Computer Engineering Department, Sarvajanik College of Engineering & Technology, Surat, Gujarat, India

* **Correspondence:** Email: vandana.joshi@scet.ac.in.

## Supplementary materials

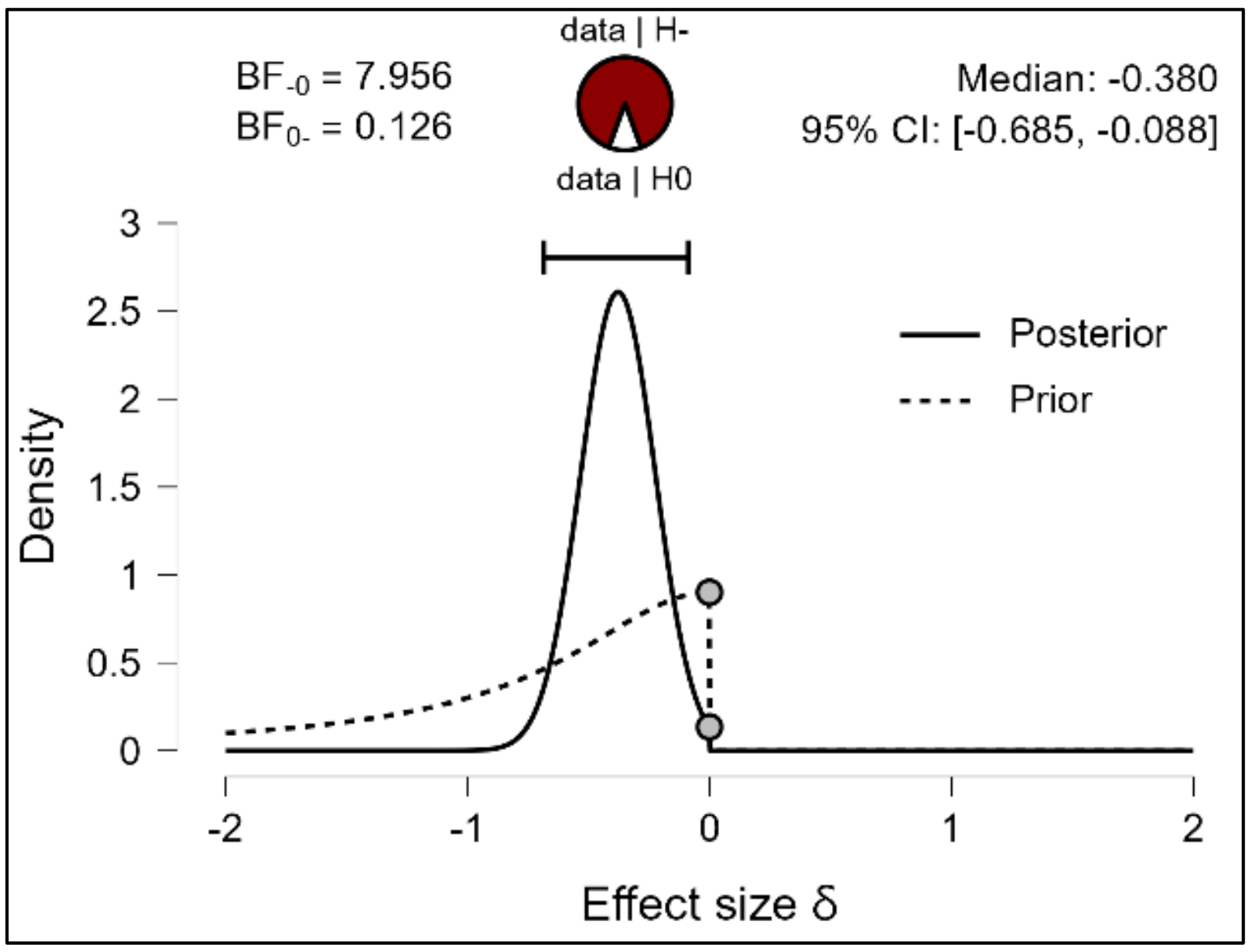


**Figure 1.** The prior and posterior plot with the Bayes Factor value for the Beta wave in the Combined subject in the EO state.

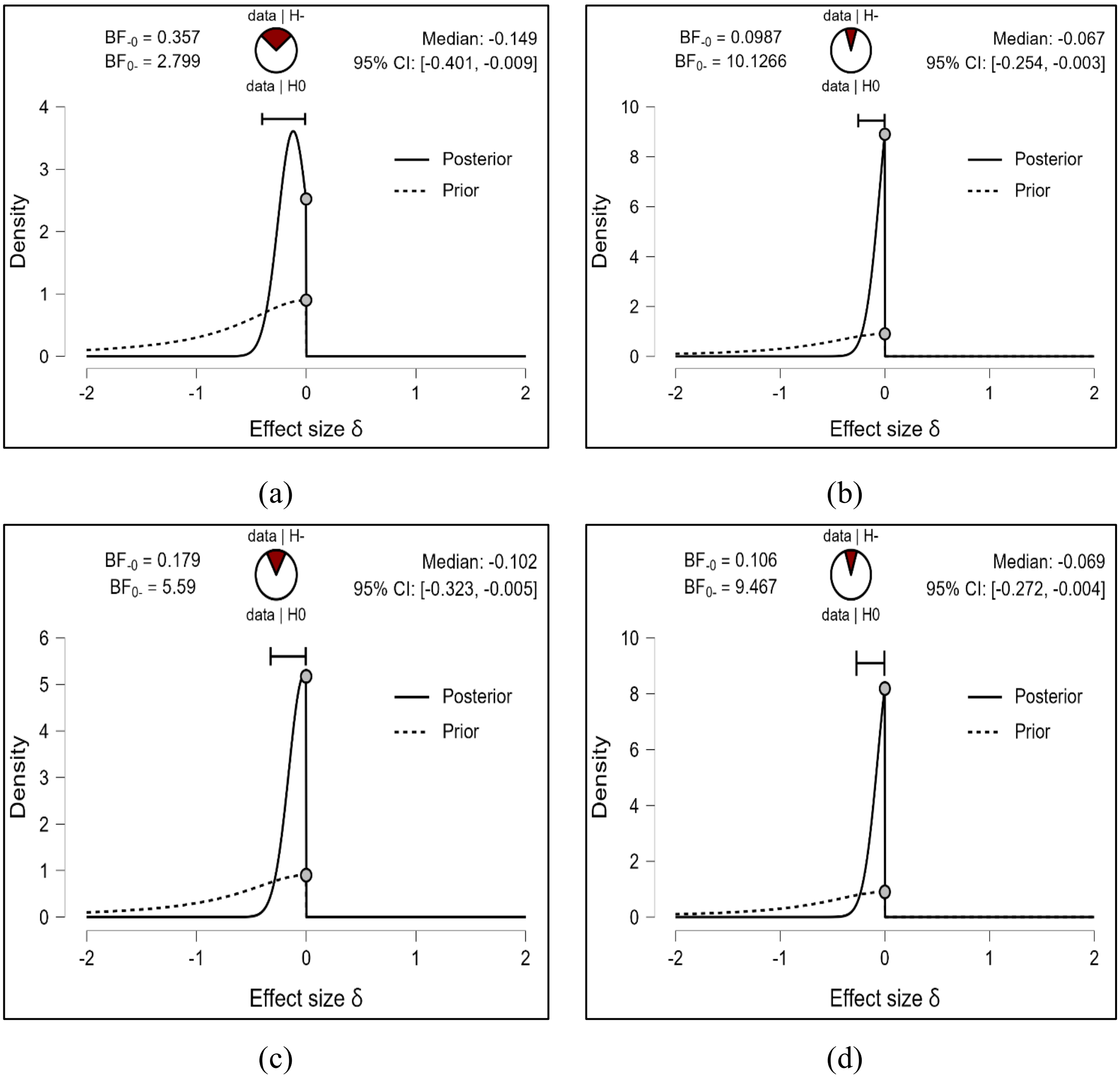


**Figure 2.** The prior and posterior plot with the Bayes Factor value for all sub-bands in the Inattentive subject in the EO state (a) Delta (b) Theta (c)Alpha (d) Beta.

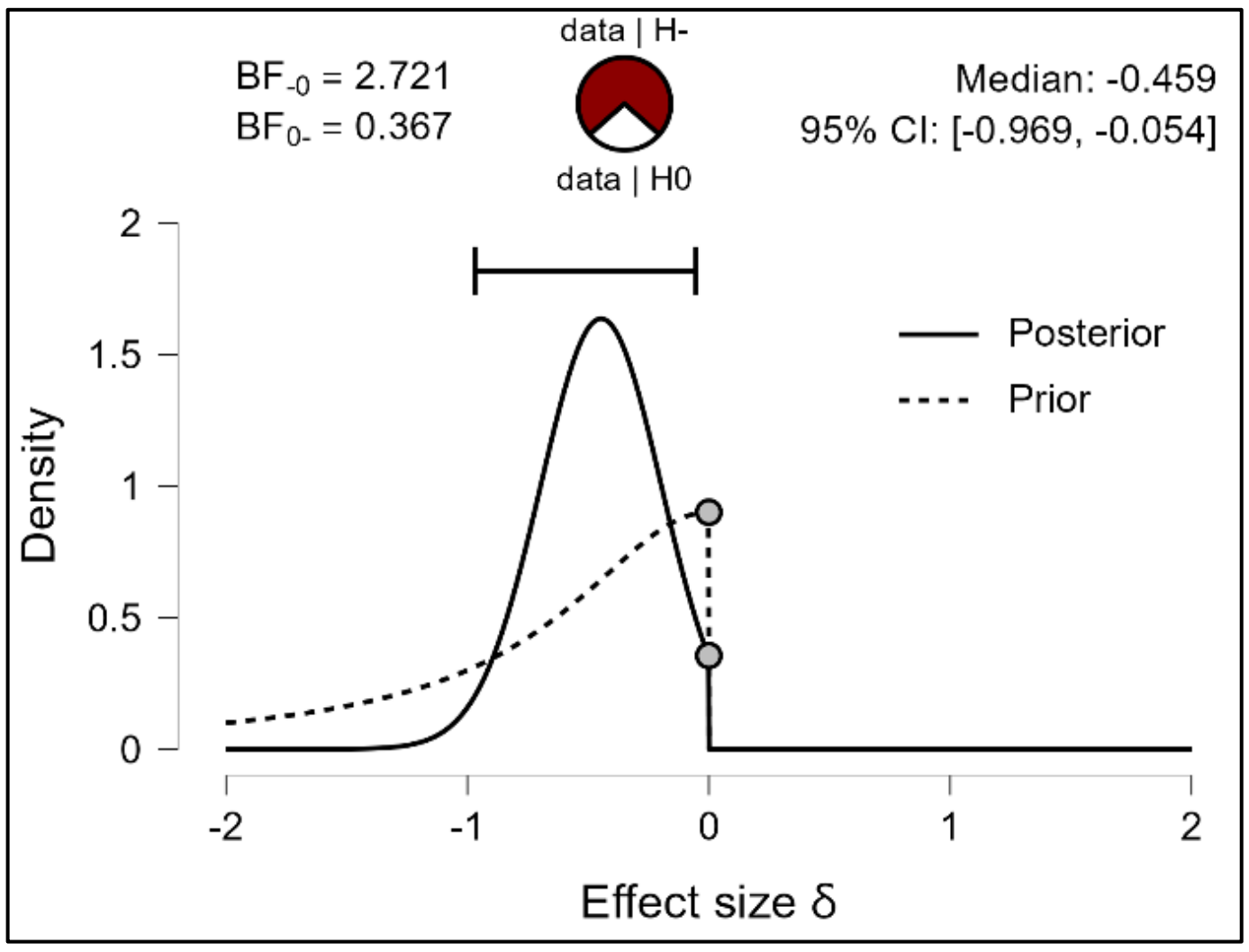


**Figure 3.** The prior and posterior plot with the Bayes Factor value for the Delta wave in the Combined subject for Dataset 3.

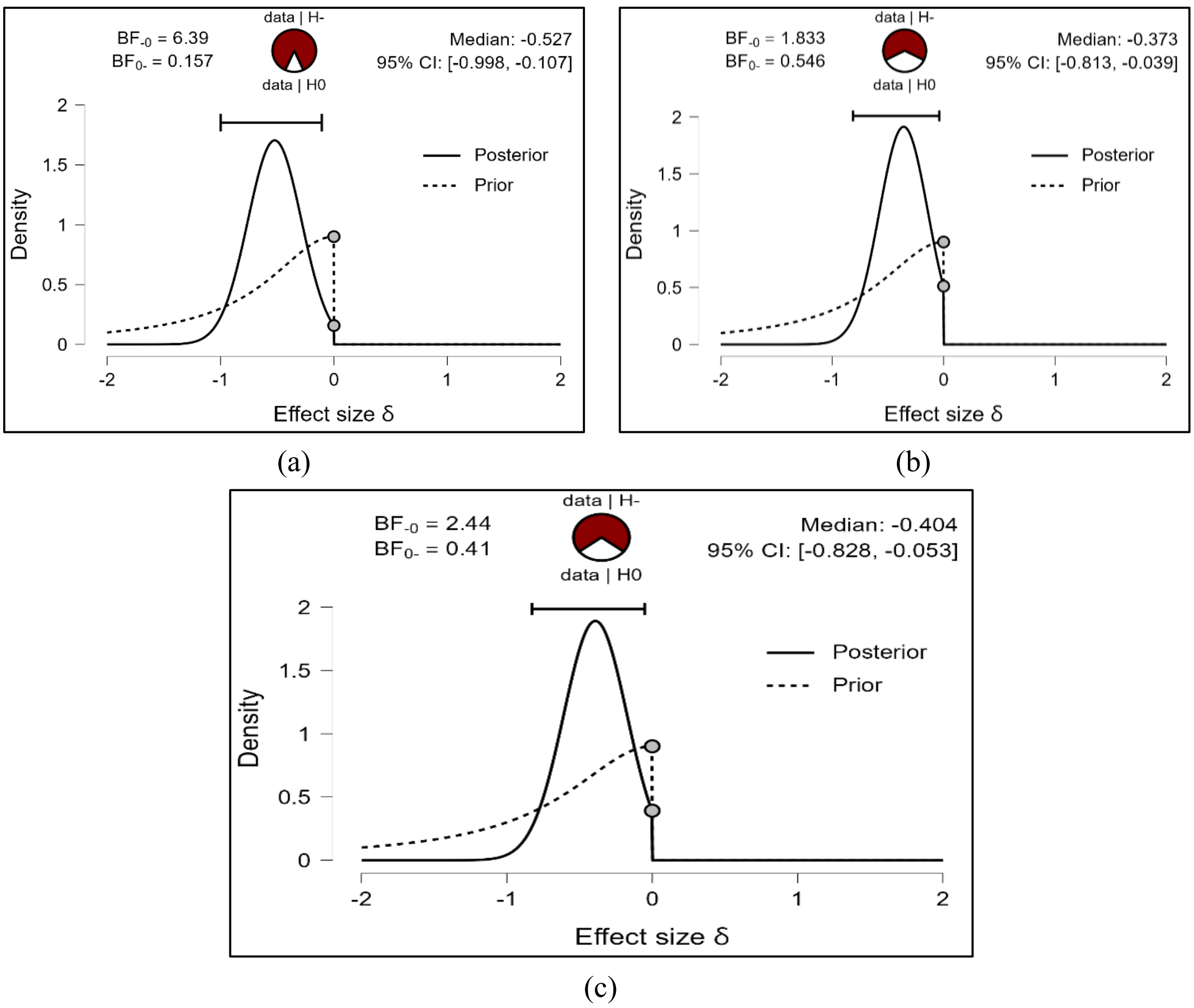


**Figure 4.** The prior and posterior plot with the Bayes Factor value for sub-bands (a) Delta, (b) Alpha, (c)Theta in the Combined subject in Dataset 4.

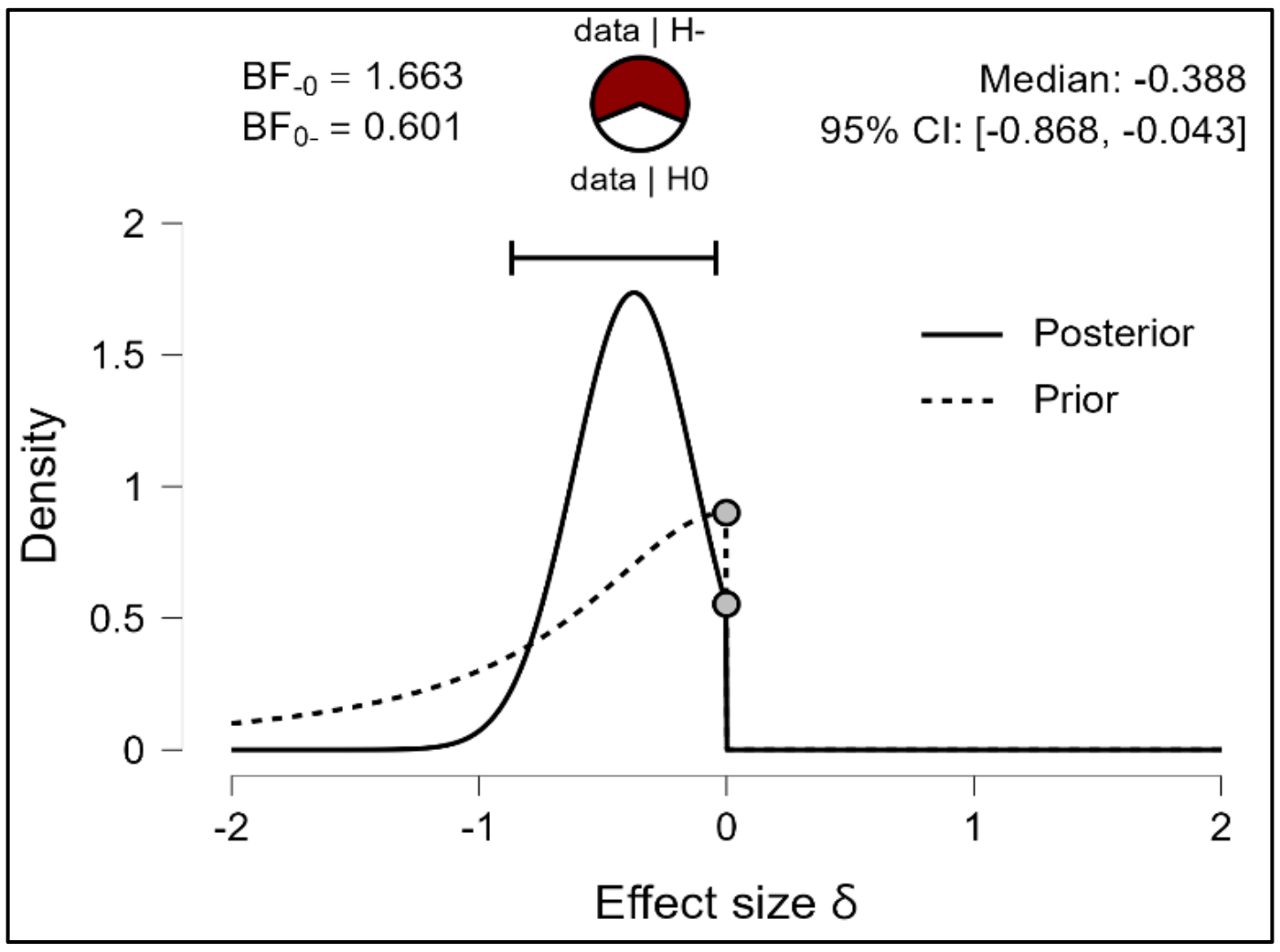


**Figure 5.** The prior and posterior plot with the Bayes Factor value for the Delta wave in the Inattentive subject for Dataset 3.

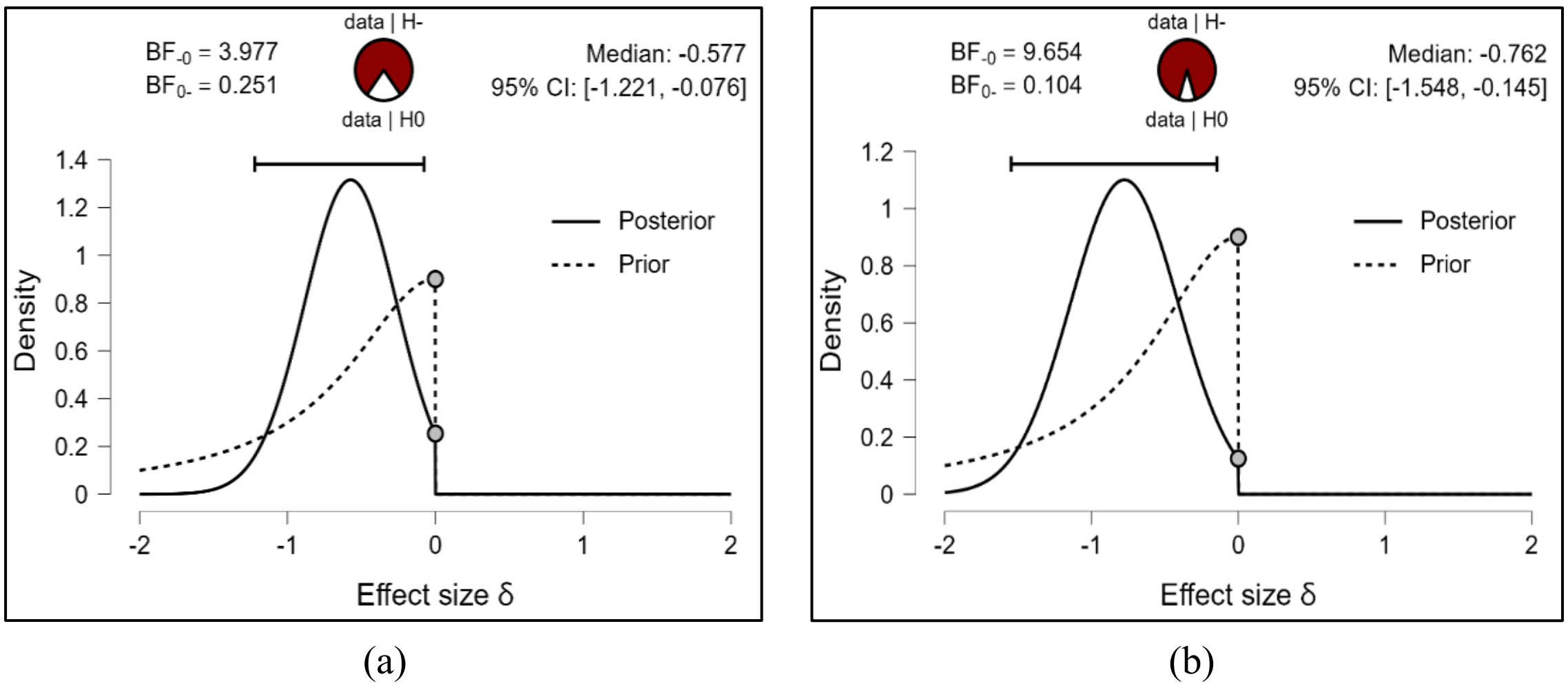


**Figure 6.** The prior and posterior plot with the Bayes Factor value for the (a) Beta (b) Alpha wave in the Inattentive subject for Dataset 4.

AIMS Press

© 2026 the Author(s), licensee AIMS Press. This is an open access article distributed under the terms of the Creative Commons Attribution License (https://creativecommons.org/licenses/by/4.0)
